# Medical care of patients with Wilson disease in Germany: a multidisciplinary survey among university centers

**DOI:** 10.1186/s13023-023-02731-4

**Published:** 2023-05-24

**Authors:** Sebastian Zimny, Hélène Bourhis, Sabine Weber, Florian Paul Reiter, Simon Hohenester, Eduard Kraft, Isabelle Mohr, Uta Merle, Karl Heinz Weiss, Gerald Denk

**Affiliations:** 1 Department of Medicine II, University Hospital, LMU Munich, Marchioninistraße 15, 81377 Munich, Germany; 2 Department of Medicine II, University Hospital Würzburg, Oberdürrbacher Straße 6, 97080 Würzburg, Germany; 3 Department of Physical Medicine and Rehabilitation, University Hospital, LMU Munich, Marchioninistraße 15, 81377 Munich, Germany; 4 Department of Internal Medicine IV, University Hospital Heidelberg, Im Neuenheimer Feld 410, 69120 Heidelberg, Germany; 5 Internal Medicine, Salem Medical Center, Zeppelinstraße 11 – 33, 69121 Heidelberg, Germany; 6 Transplant Center, University Hospital, LMU Munich, Marchioninistraße 15, 81377 Munich, Germany

**Keywords:** Wilson disease, Medical care, Surveillance

## Abstract

**Background:**

Wilson disease (WD) is a rare, hereditary disorder of copper metabolism. Due to its variable symptoms and manifestations, diagnosis remains challenging. Affected patients must obtain lifelong medical treatment, as the disease is fatal if untreated. Patients require continuous monitoring, but little is known about the care of these patients in Germany. Therefore, we analyzed the medical care of WD patients at German university centers. We sent a questionnaire containing 20 questions to a total of 108 departments of pediatrics, neurology and gastroenterology in 36 university hospitals. Our questions referred to the characteristics of WD patients at the different sites and internal procedures regarding diagnosis, therapy and follow-up. A descriptive statistical analysis was performed.

**Results:**

Sixty-three departments (58%) returned our questionnaire. In total, approximately one-third of the estimated WD patients in Germany are seen annually in the outpatient clinics of these departments (approx. 950 patients). There are only a few departments which treat patients in a multidisciplinary setting (12%). Our survey revealed that for diagnosis, 51% of all departments used an algorithm based on the Leipzig score as recommended by international guidelines. Most departments apply essential parameters recommended by WD guidelines. Routine monitoring is performed at least biannually by 84% of the departments, and standard investigations for monitoring are regularly applied. A routine family screening is performed by 84% of all departments. A reduction in medical therapy during pregnancy is recommended by 46% of the departments. Only 14% suggested that WD patients should not breastfeed. Liver transplantation (LT) due to WD is a rare but repeatedly occurring event. Most departments of gastroenterology (72%) reported at least one patient with LT within the last decade.

**Conclusions:**

Medical care of WD patients at German university centers follows the recommendations set forth by international guidelines, but only a few centers treat significant numbers of patients. The surveillance of patients does not follow specified standards, but most departments adhere to the accepted guidelines. The formation of central units and networks in a multidisciplinary setting should be evaluated to improve the care of WD patients.

**Supplementary Information:**

The online version contains supplementary material available at 10.1186/s13023-023-02731-4.

## Background

Wilson disease (WD) [1] is a disorder of copper metabolism mediated by autosomal recessive inherited mutations of the *ATP7B* gene on chromosome 13q. More than 700 different mutations in this gene causing WD have been described [2]. Mutations lead to copper transporter dysfunction with subsequent copper deposition in the liver and other organs. The most frequent manifestations encompass copper deposition in the cornea (Kayser–Fleischer rings) [3–5], hepatic abnormalities including acute liver failure and liver cirrhosis [6–8], and impairment of the central nervous system with neurologic manifestations including tremor and ataxia [9] as well as psychiatric symptoms. Less common features, such as (cardio-)myopathy [10, 11], renal abnormalities [12–14], hemolytic anemia [13, 15–17] and pancreatitis [18], have also been described. The disease can manifest at any age, with a majority of patients being diagnosed between 5 and 35 years of age (mean: 13 years of age) [19–21]. The estimated prevalence of WD is approximately 1:30,000 [22, 23], occurring in approximately three thousand patients in Germany (estimated population in Germany on 31 Dec 2021: 83.2 million; data source: The Federal Statistical Office of Germany, http://www.destatis.de/; accessed on 01 Aug 2022).

Lifetime therapy and regular monitoring are necessary to avoid the otherwise progressive copper overload and subsequent fatal outcome of this disease [20]. Copper removal as well as prevention of reaccumulation are achieved by copper chelators, e.g., D-penicillamine (DPA) as a first-line drug or trientine. Zinc salts, which interfere with intestinal copper absorption, are also applied in patients [20, 21]. Further strategies include dietary recommendations to avoid copper-rich dietary components [21]. Liver transplantation (LT) is performed in cases of acute liver failure (ALF) or decompensated liver cirrhosis [20, 21].

Several guidelines for WD management have been issued, e.g., by the American Association for the Study of Liver Diseases (AASLD) in 2003, 2008 [24] and 2022 [21], by the European Association for the Study of the Liver (EASL) in 2012 [20] and by the European Society for Pediatric Gastroenterology, Hepatology and Nutrition (ESPGHAN) in 2018 [25]. German guidelines were published by the German Society for Neurology in 2012 [26].

Whereas the medical care of highly prevalent liver diseases such as nonalcoholic fatty liver disease (NAFLD) has been described in Germany [27], data on the medical care of patients with orphan liver diseases are scarce. Therefore, in this study, we analyzed the medical care of WD patients in Germany.

## Results

### Medical discipline distribution, WD prevalence and multidisciplinary approaches

We sent our questionnaire, comprising 20 questions, to departments of pediatrics, neurology and gastroenterology/hepatology in 36 German university hospitals (108 departments in total). Fifty-eight percent (n = 63/108) of the 108 departments returned the questionnaire (Additional file 1: Material 1). Six other departments briefly stated that they take care of only very few to no WD patients without returning our questionnaire. These six departments were not considered for our evaluation.

The participating centers were divided equally between the three disciplines: 35% departments of pediatrics (n = 22/63), 32% departments of neurology (n = 20/63) and 33% departments of gastroenterology (n = 21/63).

Most departments (88%, n = 53/60) stated that WD patients are not treated in a multidisciplinary outpatient clinic at their site. Only seven departments reported having a multidisciplinary approach for WD. The four departments with the highest numbers of WD patients in their outpatient clinic reported treating patients in multidisciplinary settings, while only three (5%) of the other departments had such arrangements.

**Fig. 1:**
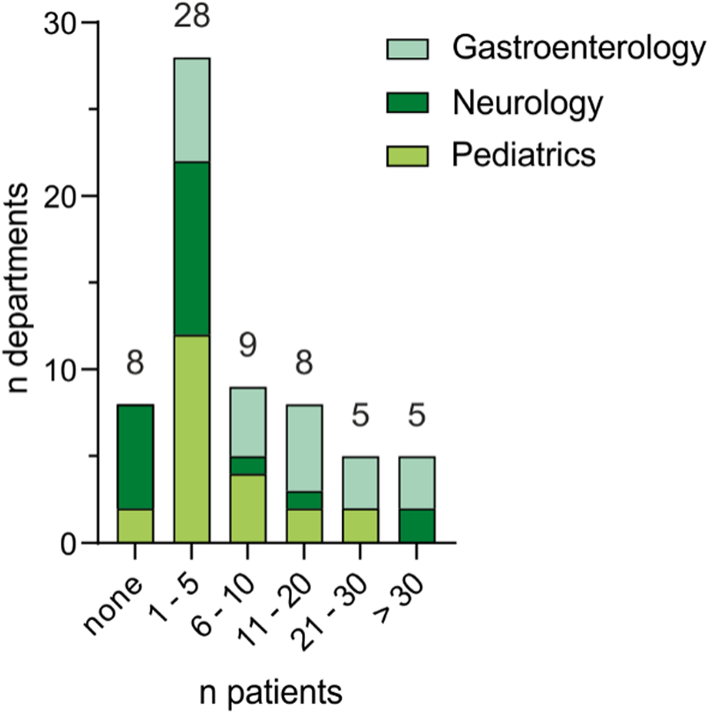
Number of patients with Wilson disease (WD) treated annually in outpatient clinics at German university centers subdivided into medical fields. Sixty-three departments provided data on their outpatient clinic. Most departments returning our questionnaire reported treating no (n = 8/63, 13%) or up to five (n = 28/63, 44%) WD patients in their outpatient clinic annually. Five departments reported between 21 and 30 patients, and five further departments reported more than 30 patients per year

The number of WD patients seen annually in each center varied relevantly between the participating departments (median (Mdn) of patients: 5; interquartile range (IQR): 2–15). Five departments saw more than 30 patients per year, whereas most departments had much lower numbers of patients in their care (Fig. 1). In total, the number of WD patients seen in the outpatient clinic was approximately 950 in all departments (n = 63; considering that some departments (n = 7) indicated margins instead of natural numbers in our questionnaire). Assuming a WD prevalence of 1:30,000 [22, 23], this number represents approximately one-third of the estimated WD patients in Germany (as mentioned above).

The number of persons treated in an inpatient setting was expectedly lower (Mdn of patients: 1; IQR: 0–2). Thirty-three percent (n = 20/61) of the participating departments did not treat any patients in their wards annually, and 61% (n = 37/61) treated up to 5 patients (Additional file 1: Material 2).

### WD diagnosis: symptoms, age distribution, use of the Leipzig score and diagnostic investigations

**Fig. 2:**
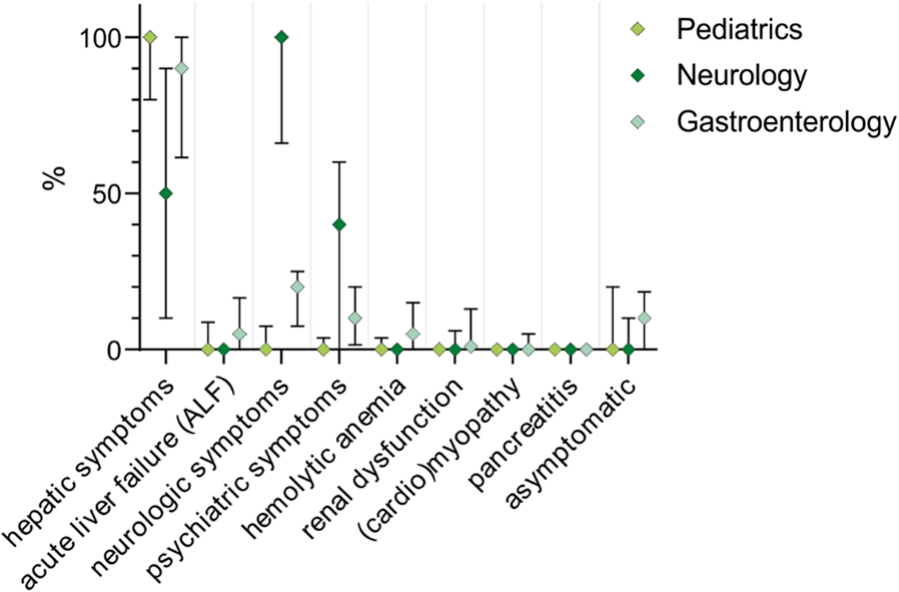
Signs and symptoms in WD patients at the time of diagnosis in German university centers, subdivided into medical disciplines. Fifty-six departments provided data on signs and symptoms (20 departments of pediatrics, 15 departments of neurology and 21 departments of gastroenterology). The percentages of patients with certain symptoms reported by the participating centers are displayed (median with interquartile range; values are shown in Additional file 1: Material 3). Most departments reported hepatic and/or neurologic manifestations at the time of diagnosis, whereas further symptoms were less common

For a better understanding of the population of WD patients in German university centers, we included questions about symptoms at diagnosis and the age of onset in our survey. We took the proportions for specific symptoms reported by the departments for our evaluation with Mdn and IQR. The results are presented as Mdn. The departments reported that in most cases, WD patients initially had hepatic symptoms or signs of hepatic damage (90% in all departments, n = 56). Not surprisingly, almost all WD patients had initial neurologic symptoms in departments of neurology (100% vs. 10% in departments of pediatrics and gastroenterology). Other manifestations had minor roles during disease onset. Asymptomatic patients were hardly described (Mdn 0% with IQR 0–16 in all departments). Fifty-six out of 63 responding departments provided data regarding symptoms at diagnosis. The proportions reported by the departments (Mdn and IQR) are shown in Fig. 2 and Additional file 1: Material 3.

Most patients were older than 18 years at the time of diagnosis, as reported by the departments on average (60%). The distribution pattern of age is shown in Additional file 1: Material 4.

**Fig. 3:**
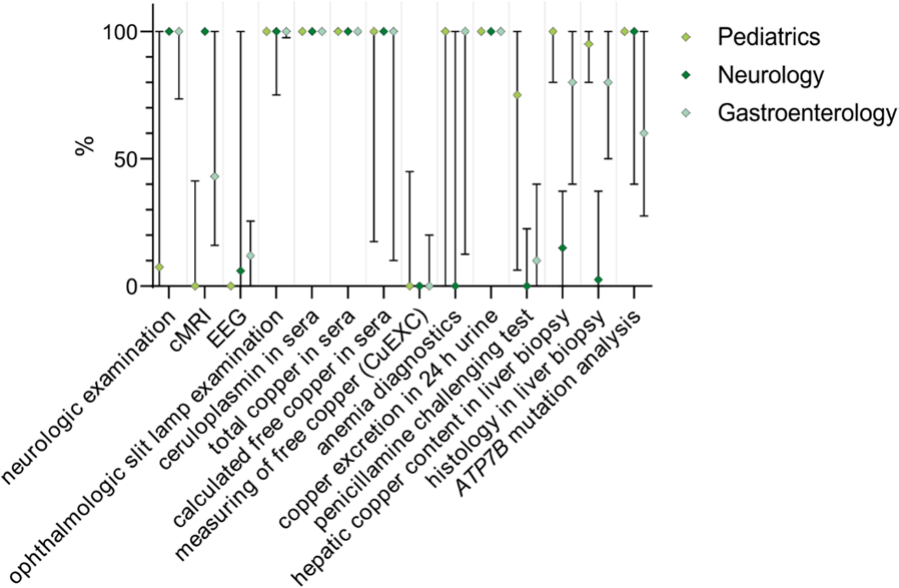
Diagnostic investigations for WD diagnosis at German university centers subdivided into medical disciplines. Fifty-five departments provided data on the use of diagnostic investigations (20 departments of pediatrics, 14 departments of neurology and 21 departments of gastroenterology). The percentages reported by the participating centers are displayed (median with interquartile range; values are shown in Additional file 1: Material 6). Most departments perform diagnostic investigations as recommended by guidelines, e.g., determination of ceruloplasmin and copper in patients’ sera as well as urinary copper excretion

The accumulation of copper evokes a heterogeneous variety of symptoms and clinical manifestations in WD patients, which often impedes a straightforward diagnosis. Guidelines recommend algorithms for diagnosis, e.g., based on the Leipzig score by Ferenci et al. [20, 21, 25, 26, 28]. Fifty-one percent (n = 29/57) of the departments reported the use of the Leipzig score [28] on a regular basis (Additional file 1: Material 5). Nevertheless, the most relevant parameters that are well established for the diagnosis of WD are determined frequently by the departments. The results are again presented as Mdn. Ceruloplasmin and copper in patients’ sera as well as urinary copper excretion were determined in almost all departments independently of the medical field (100% each). Cerebral magnetic resonance imaging (cMRI) was performed in most departments of neurology on average (100% vs. 20% in departments of pediatrics and gastroenterology). Liver biopsy for hepatic copper content was performed more often in departments of pediatrics (100%) and gastroenterology (80%) than in departments of neurology (15%). Genetic investigation (*ATP7B* mutation analysis), one out of the seven components of the Leipzig score by Ferenci et al. [28], provides the most accurate diagnosis in most cases and was used by departments of pediatrics (100%), neurology (100%) and gastroenterology (60%). Fifty-five out of the 63 responding departments provided data regarding diagnostic investigations for WD patients. The proportions of performed investigations reported by the institutions (Mdn and IQR) are shown in Fig. 3 and Additional file 1: Material 6.

### WD monitoring: frequency of monitoring visits, monitoring investigations and therapy

**Fig. 4:**
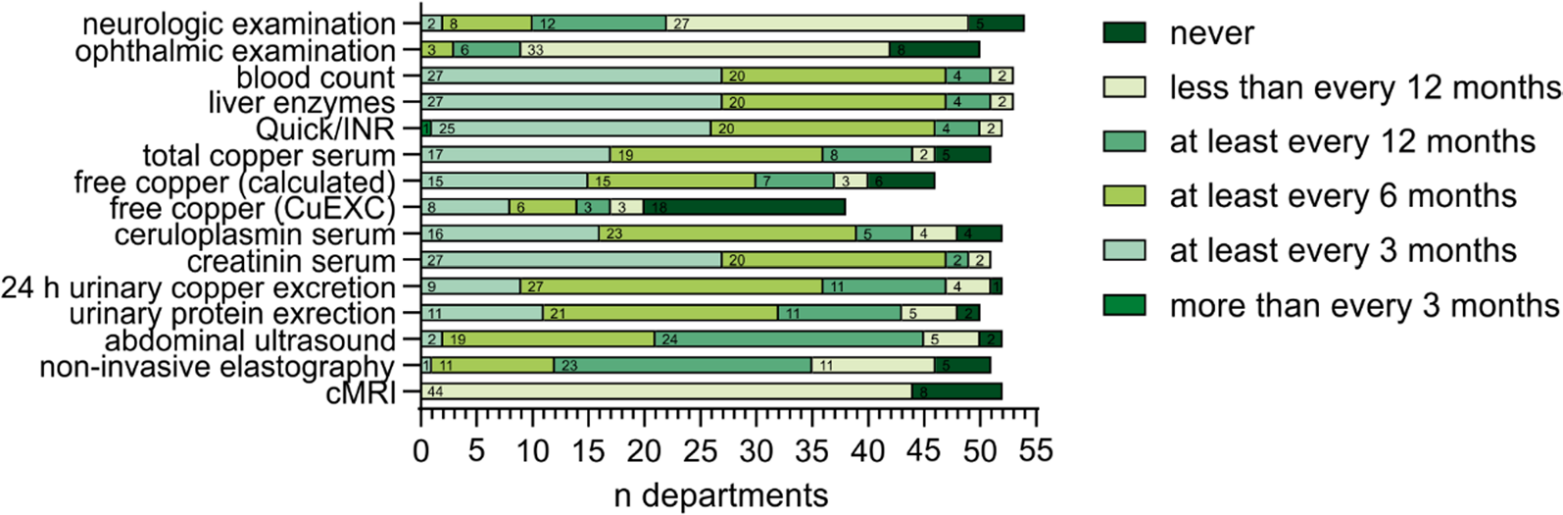
Frequency of monitoring investigations used for WD patients in German university centers. Up to fifty-four departments (depending on the procedure) provided data on their application of certain monitoring investigations. Most investigations are used at least annually or more often. The application of monitoring investigations subdivided into disciplines is shown in Additional file 1: Material 8

Regular monitoring is crucial to ensure the adequacy of long-term therapy. Based on our survey, most WD patients are seen at least every three months (45%, n = 25/55) to six months (38%, n = 21/55). Taken together, 84% (n = 46/55) of all departments follow-up with their patients at least biannually (Additional file 1: Material 7). The procedures performed during check-up varied between the departments. Total serum copper (71%, n = 36/51), ceruloplasmin (75%, n = 39/52) and 24-h urinary excretion of copper (69%, n = 36/52) as well as liver enzymes (89%, n = 47/53), international normalized ratio (INR, 88%, n = 46/52) and total blood count (89%, n = 47/53) were determined by most departments at least twice a year. Only 18% (n = 10/54) of departments performed neurologic examinations at least biannually. The frequencies of monitoring investigations performed by the participating departments are shown in Fig. 4 and Additional file 1: Material 8.

WD patients need to obtain lifelong medical treatment to prevent the reaccumulation of copper and disease progression. With adequate therapy, the prognosis for WD patients is good [29]. To monitor treatment effectiveness, guidelines recommend determining the 24-h urinary copper excretion. The European and the neurologic German WD guidelines recommend the cessation of chelator therapy two days before determination of 24-h urinary copper excretion [20, 26, 30], which is also mentioned in the American guidelines as an alternative method [21]. In our survey, 48% (n = 24/50) of the departments reported adherence to recommendations for chelator cessation before measuring 24-h urinary copper excretion (Additional file 1: Material 9). The proportion of departments that paused chelator treatment before the measurement was low in departments of pediatrics (15%, n = 3/20) and high in departments of neurology and gastroenterology (taken together 70%, n = 21/30).

Medical WD treatment with zinc salts has also been established. In our survey, less than half of the participating departments (44%, n = 23/52) declared that zinc salt monotherapy was used to treat their WD patients. Interestingly, neither the majority of departments of neurology (38%, n = 5/13) nor gastroenterology (35%, n = 7/20) reported the use of a zinc salt monotherapy on a regular basis, but many departments of pediatrics (58%, n = 11/19) regularly used zinc salt monotherapy (Additional file 1: Material 10A). Considering departments treating patients solely with a monotherapy (either chelators or zinc salts, n = 33), the mean proportions for DPA, trientine and zinc salts were 72%, 19% and 9%, respectively. Thus, most departments treated most of their patients with DPA as first-line therapy. Trientine, as second-line therapy, was used less commonly (Additional file 1: Material 10B). The application of zinc salts in combination with chelators was used by only 28% (n = 15/54) of the departments (Additional file 1: Material 10C).

Since medical treatments have side effects, a dose reduction of lifelong therapy is desirable. We therefore asked for the time point of evaluation of reduced chelator therapy in stable patients. A total of 81% (n = 43/53) of departments reevaluated the treatment of WD patients to check if a reduction in medication was possible (Additional file 1: Material 11). Most departments reassessed therapy after up to two years (49%, n = 26/53) or between two and five years (26%, n = 14/53).

### Family screening, transition programs, treatment during pregnancy and breastfeeding, nutrition counseling and liver transplantation

WD is an autosomal recessive inherited disease. A routine screening of family members was performed in 84% (n = 46/55) of the departments (Additional file 1: Material 12). A structured transitioning program from pediatrics to adult medicine was available in a fourth of departments (27%, n = 15/55).

With appropriate treatment, pregnancy is achievable for female WD patients. However, these patients require special monitoring. A dose reduction was performed by 46% (n = 11/24) of all departments. Seventeen percent (n = 4/24) of the departments reduced a chelator therapy and combined it with zinc salts, while 13% (n = 3/24) switched to zinc salt monotherapy in pregnant patients. Twenty-five percent (n = 6/24) of all participating departments stated that therapy was not adapted for pregnant patients (Additional file 1: Material 13A). Regarding breastfeeding, 23% (n = 5/22) of the departments did not adapt therapy in our survey. A dose reduction of chelator therapy was recommended by 36% (n = 8/22) of the departments. Only 14% (n = 3/22) of departments recommended that women should not breastfeed their children (Additional file 1: Material 13B). The number of departments providing statements about pregnancy and breastfeeding was low (out of a total of 63 departments, 24 and 22 departments, respectively, provided statements).

Professional nutrition education was offered in 63% (n = 34/54) of all departments (Additional file 1: Material 14).

**Fig. 5:**
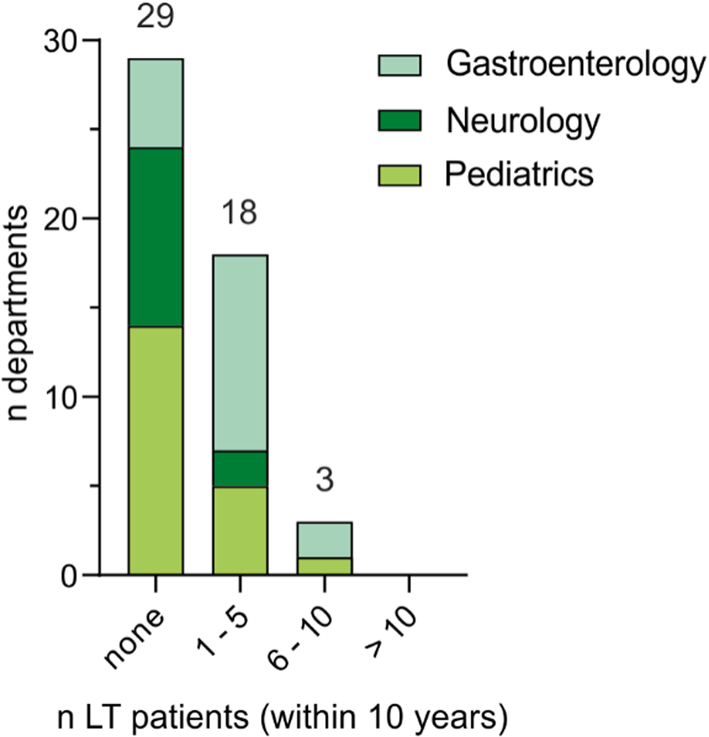
Number of patients treated in German university centers who have undergone liver transplantation (LT) within the last decade. Fifty departments provided data on LT. LT is an uncommon event in WD patients, and most departments do not have LT patients. In particular, departments of gastroenterology report patients who have undergone LT within the last decade

LT is an uncommon event for WD patients in Germany. Three departments reported having between 6 and 10 LT patients in their care, and it was an exception in most centers (Fig. 5). Nevertheless, especially in departments of gastroenterology, LT is a common event (Mdn of WD patients in departments of gastroenterology: 3; IQR: 2–5). Seventy-two (n = 13/18) of departments of gastroenterology reported treating at least one patient who underwent LT within the last decade (vs. 30%, n = 6/20, in departments of pediatrics and 17%, n = 2/12, in departments of neurology).

## Discussion

In this study, we evaluated the medical care of WD patients at German university hospitals. We sent a questionnaire to 108 departments in 36 German university centers and received the questionnaire back from 58% of the departments. The three disciplines of pediatrics, neurology and gastroenterology were distributed equally among the participating departments. The departments indicated that a total of approximately 950 patients are treated in their outpatient clinics annually, presumably covering approximately one-third of the expected WD patients in Germany.

WD mainly presents with a hepatologic or neurologic phenotype, but its symptoms can vary widely. Information about the prevalence of clinical manifestations varies heavily in the literature, e.g., regarding hepatic symptoms between 15 [31] and 84% [32]. In our survey, most centers reported a high prevalence of hepatic and/or neurologic symptoms in their patients at the time of diagnosis. Other manifestations at the time of diagnosis did not occur to any noteworthy extent. A limiting factor was the circumstance that the proportions of symptoms in the centers might be unbalanced due to varying numbers of patients in the institutions. Nevertheless, our results are in line with the literature.

Due to its manifold symptoms, its variable clinical course, and complex biochemical tests that are sometimes difficult to interpret, the establishment of a diagnosis of WD can be challenging. The use of gene panels in groups with certain phenotypes could provide a quicker diagnostic approach but is often not applied in the first place, and inconclusive and often complex biochemical test results can delay diagnosis. In particular, patients with neurologic manifestations experience longer time periods between the onset of symptoms and diagnosis, with a delay of up to several years [33]. Guidelines recommend the use of algorithms for diagnosis, e.g., based on the Leipzig score by Ferenci et al. [20, 21, 25, 26, 28]. In contrast, the proportion of departments using the Leipzig score was markedly low; only 51% of all responding departments indicated its use in their clinical routine. Nevertheless, the most relevant parameters of the Leipzig score were used for diagnostics by most departments on a regular basis. Serum ceruloplasmin, serum copper and copper excretion in 24-h urine were used by almost all departments to establish the diagnosis of WD. The determination of unbound copper, referred to as “free copper” or nonceruloplasmin-bound copper concentration (NCC) [34], was less common. Usually, NCC is not measured directly but is calculated from total serum copper and ceruloplasmin [35]. A rather new method of copper evaluation is the direct determination of exchangeable copper (CuEXC) [36], which has been proposed to have a very high sensitivity and specificity for WD [37]. CuEXC has not yet been implemented in the European guidelines, but is mentioned, e.g., in the American AASLD and pediatric ESPGHAN guidelines, as a promising and valuable monitoring parameter [21, 25]. In our survey, most departments stated that CuEXC was not determined. CuEXC determination may become more relevant after implementation in recent guidelines. It must be taken into account, however, that the difference between CuEXC (measured free copper) and NCC as calculated free copper might not be obvious in all laboratories or understood by all survey participants. Additionally, the actual number of laboratories determining CuEXC might diverge.

The European EASL guidelines as well as the American AASLD guidelines recommend regular routine monitoring, at least biannually [20, 21]. The pediatric ESPGHAN guidelines state that children should be monitored every 3–6 months after the initial therapy and remission phase [25]. Longer intervals between monitoring visits are proposed by the neurologic German guidelines, which state that routine monitoring should be carried out every one to two years [26]. On average, 84% of the participating departments indicated utilizing a monitoring interval of 6 months or less. We therefore conclude that monitoring is performed in adequate intervals, as recommended by international guidelines. The following investigations are recommended for routine monitoring [21, 25, 26]: physical and neurologic examination, serum copper and ceruloplasmin, liver enzymes and INR, complete blood count and urine analysis. In addition to rarely performing neurologic examinations during routine monitoring, most departments performed these monitoring components frequently. Only the neurologic German guidelines recommend performing a cMRI every 4–6 years to detect latent changes [26], whereas the American guidelines state that repeated cMRI is not useful in general [21]. All departments stated that they performed cMRI less than once a year (n = 44/52) or never (n = 8/52) in routine monitoring.

In addition to an early diagnosis, effective medical therapy is another key factor affecting the prognosis of WD patients. Treatment takes place in two phases: after the initial phase of removing and detoxifying accumulated tissue copper, the goal of lifelong medical therapy is to prevent copper reaccumulation and disease progression. If treatment is initiated early and adherence is high, the prognosis for WD patients is good, and life expectancy is normal [29]. The only reason to terminate pharmaceutical therapy in WD is a history of LT. Medical therapy encompasses copper chelators, such as DPA and trientine. The European and German guidelines recommend the monitoring of chelator treatment adequacy for both DPA and trientine by measuring the 24-h urinary copper excretion, while on treatment, two days after chelator cessation [20, 26]. Cessation of chelator therapy was reported by 48% of all departments. In line with heterogeneous guideline statements, the proportion of departments interrupting chelator therapy was low in departments of pediatrics (15%) and high in departments of neurology/gastroenterology (70%). In addition to chelator therapy, treatment with zinc salts (e.g., sulfate, acetate, gluconate) to block enteral copper absorption has been established since the early 1960s [20, 21, 38, 39]. Zinc therapy may be less effective than chelator therapy [40] but may be used for maintenance therapy as well as for asymptomatic or presymptomatic patients, especially in pediatric patients [20, 41]. Zinc can be used as monotherapy or combined with chelators [20, 21, 26]. Zinc monotherapy can be effective and safe in WD patients with neurologic manifestations, but should be used cautiously in cases of hepatic manifestation because of potential hepatic deterioration [20, 21]. Due to having a better tolerance profile with fewer side effects, zinc salt therapy has become more popular, especially in departments of pediatrics [20, 21, 25, 41]. In our survey, 43% of the departments stated that zinc salts are used for WD monotherapy. Fifty-eight percent of the departments of pediatrics reported the use of zinc salt monotherapy. It is likely that the need to treat patients with better tolerated medication is higher in the pediatric setting. Zinc salts in combination with chelators are used by a minority of departments (28%). On average, departments used the first-line drug DPA in 72% and the second-line drug trientine in 19% of their patients.

In this context, the AASLD guidelines suggest lower chelator dosages or a shift to zinc monotherapy for stable patients, indicating that these patients have usually already been treated for 1–5 years [21]. The liver function of WD patients usually normalizes after 1–2 years of treatment [20]. The improvement of symptoms in neurologic WD patients might be slower and still noticeable after 3 years of DPA treatment [42]. Most departments (81%) reevaluate the initial treatment of WD patients to check if a reduction of medication is possible, usually after two years (49%) or between two and five years (26%) after starting therapy.

In WD, most asymptomatic patients are detected by family screening [20]. Since genetic testing has become more available and affordable, guidelines suggest genetic screening for members of a WD patient´s family [20, 21, 26]. Family screening was recommended by most departments (84%) in our survey. We emphasize the need to perform family screening to identify asymptomatic patients.

A structured transitional program from pediatrics to adult medicine was available in a fourth of departments (27%). Regarding the necessity of lifelong therapy, the need for good monitoring and the risk of noncompliance [43], transition programs can be helpful.

Guidelines provide recommendations regarding pregnancy and breastfeeding. As there is an increased risk of developing WD among the children of WD patients, genetic counseling and haplotype analysis of patients´ partners is recommended [20]. Despite reports of teratogenic effects, guidelines declare that treatment should be continued throughout pregnancy [20, 21, 26]. Cessation of therapy can lead to clinical deterioration, including ALF and spontaneous abortion [21, 44, 45]. Due to possible teratogenicity, chelator reduction is recommended at the beginning of pregnancy as early as possible [20, 21]. A reduced chelator dose is also recommended for the last trimester to prevent copper deficiency in the unborn child as well as to improve wound healing in the mother in case of cesarean section [21, 26]. To our surprise, the recommended dose reduction was carried out in only 46% of all departments. Twenty-five percent of all departments stated that they do not make therapy adjustments during pregnancy. Breastfeeding is not recommended during chelator therapy because of drug excretion into breast milk, although reports suggest that there is no harm for children of breastfeeding mothers under chelator therapy [20, 26, 46]. The American guidelines propose that the pros and cons of breastfeeding should be discussed with patients individually [21]. In our study, only 14% of all departments stated that women were advised to avoid breastfeeding. Although the number of departments answering our questionnaire about procedures for women with WD was low and the power of statements might be limited, we assume, due to the inconsistency of answers, that more studies should be conducted for WD in pregnant or breastfeeding women.

Some foods contain high levels of copper, such as shellfish, chocolate/cocoa and mushrooms [21, 26]. Guidelines recommend the avoidance of these dietary components, at least in the first year of treatment [20, 21, 26]. In this context, professional nutrition counseling is offered in 63% of the departments of our survey.

In cases of ALF or progressive, decompensating liver cirrhosis, LT may be the only therapeutic option. WD is the primary indication for 1% of all LTs in Europe. WD is the underlying disease in up to 12% of all ALF patients who are listed for emergency LT [20, 21, 47]. In 2021, 834 LTs were performed in Germany. Five patients (0.6%) had WD (data source: Eurotransplant, https://www.eurotransplant.org; data provided on 18 Aug 2022 upon request req235.2022). Although LT is a rare event for WD patients, 72% of the departments of gastroenterology reported at least one patient at their site who has undergone LT within the last decade.

Taken together, the medical care of WD patients in Germany follows recommendations by international guidelines. Nevertheless, there are only a few institutions which utilize a multidisciplinary approach. Many departments have only a few patients with WD. Certain recommendations are applied inconsistently, such as the cessation of chelator therapy before determination of 24-h urinary copper excretion. We therefore propose the establishment of larger, multidisciplinary centers to improve the medical care of WD patients.

The present study had some limitations. We analyzed the medical care of patients with WD using a retrospective approach. We received data from 63 departments (return rate: 58%). It is unclear whether other departments did not participate due to a lack of patients, interest, time or other reasons. Therefore, we must accept the risk of potential bias in our survey. We sent the questionnaire to the departments (head of department or person with managerial responsibility), but due to data protection issues, the questionnaires were completed anonymously. We do not know who filled out the survey. Nevertheless, we expect that the head of department filled out the questionnaire or that a delegated expert assistant answered the questions. The data we received might, at least in part, rely on rough estimates. Patients could be seen by different specialties simultaneously, but due to data privacy and logistical reasons, the listing of patients by name to detect the number of patients who simultaneously attend different departments would not have been feasible. A relevant number of departments returned the survey but did not answer all questions, especially if the number of patients was low. Nevertheless, we are convinced that our study adequately enables the analysis of the medical situation for patients with WD at German university centers.

## Conclusion

WD is a rare disease with limited epidemiologic data available. Our study aimed to improve knowledge about medical care at German university centers. A substantial number of 63 departments of pediatrics, neurology and gastroenterology participated in our study, covering the care of approximately 950 WD patients in their outpatient clinics. The medical care of WD patients is adequate regarding frequency and monitoring investigations, but there are only a few centers in Germany with larger numbers of WD patients. Due to low patient numbers, there might be an imbalance of expertise in the care of WD. Although there seems to be no uniform standard for the diagnosis and treatment of WD, most departments follow the recommendations of the available guidelines. The formation of larger and multidisciplinary units could improve the care of WD patients. In summary, the medical care of WD patients in Germany seems to be good. This study encourages the evaluation of medical care in a larger and international cohort and can delineate the basis for such a project.

## Methods

### Data acquisition

The data acquisition was carried out with the participation of departments of pediatrics, neurology and gastroenterology (hepatology, respectively) at German university hospitals. The acquisition was performed by a standardized questionnaire with 20 questions about the medical care of patients with WD as well as internal procedures (original questions and translation: Additional file 1: Materials 15 and 16). In October 2021, we sent the questionnaire by post to the administrative offices of 108 departments in 36 institutions. A self-addressed, stamped envelope was included to facilitate a straightforward response. We sent a reminder letter to departments that had not replied until this point in January 2022. Institutions that did not reply after an additional phone call were rated as drop-outs.

### Questionnaire

The original questions are shown in Additional file 1: Material 15. The questionnaires were marked with a continuous number (1–108). The aim was to evaluate the medical care in the participating institutions but not the individual data of patients.

The first set of questions (questions 1–4) were about the medical field of the responding institution (departments of pediatrics, neurology or gastroenterology/hepatology), the existence of a multidisciplinary outpatient clinic and the total number of WD patients attending the department per year (both outpatient or inpatient setting).

The second part (questions 5–8) addressed the diagnosis of WD: we asked for the proportion of patients with specific symptoms at the time of diagnosis, the age distribution, the use of the Leipzig score by Ferenci et al. [28] for diagnosis and the diagnostic methods most commonly used in the particular department.

A third set of questions (questions 9–14) dealt with the current monitoring of patients in the maintenance phase of the disease, when initial copper removal has been achieved and the reaccumulation of copper is adequately prevented. The questions included the frequency of visits on average, the examinations/tests carried out during monitoring visits and the medical treatment.

The fourth part of the questionnaire (questions 15–20) regarded further details such as the screening of family members, structured transition programs from pediatric to adult medicine, medicinal treatment during pregnancy and breastfeeding as well as the supply of professional nutrition counseling and the number of patients who have undergone LT within the last decade.

### Data analysis

The questionnaires we received were collected, and the data were transferred into SPSS software (Version 28, IBM, Armonk, NY, USA). Visual presentation of the results was performed with GraphPad Prism (Version 8, GraphPad Software, Inc., San Diego, CA, USA). Missing values were not included in the analysis. If a department stated ranges instead of natural numbers, the mean value was used for analysis. Inconclusive data were excluded from the evaluation. Because of the questionnaire’s design, a descriptive statistical analysis was performed without calculating statistical significance. The percentages are calculated as the proportion of specific answers to all responding departments of the individual question; therefore, the total number of departments changes between questions.

## Supplementary Information

**Additional file 1: Material 1.** German university centers. Schematic map of Germany containing all centers that received our questionnaire (n = 108). The responding departments (n = 63/108, 58%) are highlighted by color for each discipline. **Material 2.** Number of patients with Wilson disease (WD) treated annually in an inpatient setting at German university centers subdivided into medical fields. Sixty-one departments provided data on the inpatient setting. Most departments returning our questionnaire treat no (n = 20/60, 33%) or up to five (n = 37/61, 61%) WD patients in an inpatient setting annually. Four departments reported between 6 and 10 patients annually. **Material 3.** Median and interquartile range (IQR; if applicable) for signs and symptoms in WD patients at the time of diagnosis in German university centers, subdivided into medical disciplines, as presented in Fig. 2. The IQR is shown as a range from Q1 (25%) to Q3 (75%). **Material 4.** Age distribution of WD patients at the time of diagnosis. Fifty-two departments provided data on age distribution (18 departments of pediatrics, 14 departments of neurology and 20 departments of gastroenterology). The majority (60%) of patients were older than 18 years at the time of diagnosis. In departments of neurology, 62% of patients were older than 35 years. **Material 5.** Application of the Leipzig score for WD diagnosis. Fifty-seven departments provided data on the Leipzig score (20 departments of pediatrics, 16 departments of neurology and 21 departments of gastroenterology). Fifty-one percent (n = 29/57) of all departments apply the Leipzig score. The proportion is higher in departments of gastroenterology (67%) than in departments of pediatrics (45%) or neurology (38%). **Material 6.** Median and interquartile range (IQR; if applicable) for diagnostic investigations for WD diagnosis at German university centers subdivided into medical disciplines, as presented in Fig. 3. The IQR is shown as a range from Q1 (25%) to Q3 (75%). **Material 7.** Frequency of routine monitoring for WD patients. Fifty-five departments provided data on the frequency of monitoring (20 departments of pediatrics, 14 departments of neurology and 21 departments of gastroenterology). Eighty-four percent (n = 46/55) of all departments see their patients at least biannually. Monitoring intervals tend to be shorter in departments of pediatrics (80% every three months, n = 16/20) than in departments of neurology (57% every 12 months or less, n = 8/14). **Material 8.** Frequency of monitoring investigations for WD patients in German university centers subdivided into disciplines. Up to 20 departments of pediatrics (**A**), 14 departments of neurology (**B**) and 21 departments of gastroenterology (**C**) provided data on their application of certain monitoring investigations, depending on the procedure. Most investigations are performed at least annually or more often. **Material 9.** Cessation of chelator therapy before determination of 24-h urinary copper excretion for monitoring in WD patients. Fifty departments provided data on the cessation of chelator therapy (20 departments of pediatrics, 10 departments of neurology and 20 departments of gastroenterology). Forty-eight percent (n = 24/50) pause the chelator therapy before the determination. The proportion was higher in departments of neurology and gastroenterology (together 70%, n = 21/30) than in pediatrics (15%, n = 3/20). **Material 10.** Medical therapy for WD patients. (**A**) Fifty-two departments provided data on zinc salt monotherapy (19 departments of pediatrics, 13 departments of neurology and 20 departments of gastroenterology). Zinc salt monotherapy is applied by 44% (n = 23/52) of all departments. The proportion was higher in departments of pediatrics (58%, n = 11/19) than in departments of neurology (38%, n = 5/13) and gastroenterology (35%, n = 7/20). (**B**) Thirty-three departments reported the usage of monotherapies alone (15 departments of pediatrics, 6 departments of neurology and 12 departments of gastroenterology). Regarding the use of chelator or zinc salt monotherapy in the departments, the mean in the departments was highest for D-penicillamine (72%). Trientine was less common (19%), and zinc salt monotherapy was rarely used (9%). (**C**) Fifty-four departments provided data on combination therapies (20 departments of pediatrics, 13 departments of neurology and 21 departments of gastroenterology). Zinc salt combination therapy was reported by 28% of the departments (n = 15/54). **Material 11.** Time point of chelator therapy reevaluation in WD patients. Fifty-three departments provided data on therapy reevaluation (19 departments of pediatrics, 13 departments of neurology and 21 departments of gastroenterology). Eighty-one percent (n = 43/53) reevaluated the therapy, mostly after up to two years (49%, n = 26/53) or between two and five years (26%, n = 14/53). The evaluation of a chelator dose reduction after up to 2 years was more frequent in departments of pediatrics than in those of neurology and gastroenterology (58%, n = 11/19 vs. 44%, n = 15/34 in departments of neurology and gastroenterology taken together). **Material 12.** Family screening for relatives of WD patients. Fifty-five departments provided data on family screening (20 departments of pediatrics, 14 departments of neurology and 21 departments of gastroenterology). Eighty-four (n = 46/55) of all departments perform family screening on a regular basis. The proportion was lower in departments of neurology (50%, n = 7/14) than in departments of pediatrics and gastroenterology (taken together 95%, n = 39/41). **Material 13.** Recommendations for WD patients during pregnancy (**A**) and breastfeeding (**B)**. Twenty-four departments provided data on recommendations during pregnancy, and twenty-two departments provided recommendations during breastfeeding. Forty-six percent (n = 11/24) recommended a dose reduction during pregnancy, and 36% (n = 8/22) recommended a dose reduction during breastfeeding. In 25% (n = 6/24) and 23% (n = 5/22) of departments, respectively, there are no adjustments. Fourteen percent (n = 3/22) advised their patients to avoid breastfeeding. **Material 14.** Nutrition counseling for WD patients. Fifty-four departments provided data on nutrition counseling (20 departments of pediatrics, 13 departments of neurology and 21 departments of gastroenterology). A total of 63% (n = 34/54) of departments offer nutrition counseling for WD patients in their departments. The proportion was higher in departments of pediatrics (85%, n = 17/20) and gastroenterology (62%, n = 13/21) than in departments of neurology (31%, n = 4/13). **Material 15.** Original questionnaire. **Material 16.** Translation of the original questions. **Material 17.** Institutions participating in our survey, listed alphabetically, by medical discipline.

## Data Availability

The datasets used and analyzed during the current study are available from the corresponding author on reasonable request.

## Abbreviations

AASLD: Association for the Study of Liver Diseases
ALF: Acute liver failure
cMRI: Cerebral magnetic resonance imaging
CuEXC: Exchangeable Copper
DPA: D-penicillamine
EASL: European Association for the Study of the Liver
ESPGHAN: European Society for Pediatric Gastroenterology, Hepatology and Nutrition
IQR: Interquartile range
INR: International normalized ratio
LT(s): Liver transplantation(s)
Mdn: Median
NAFLD: Nonalcoholic fatty liver disease
NCC: Nonceruloplasmin-bound copper concentration
WD: Wilson disease
