# Supplementary material for: Medical care of patients with Wilson disease in Germany: a multidisciplinary survey among university centers
Source: Orphanet J Rare Dis. 2023 May 24;18:122. doi: 10.1186/s13023-023-02731-4 (PMC10207775; doi:10.1186/s13023-023-02731-4)
Supplement: Supplementary file 1 — Additional file 1: Material 1. German university centers. Schematic map of Germany containing all centers that received our questionnaire (n = 108). The responding departments (n = 63/108, 58%) are highlighted by color for each discipline. Material 2. Number of patients with Wilson disease (WD) treated annually in an inpatient setting at German university centers subdivided into medical fields. Sixty-one departments provided data on the inpatient setting. Most departments returning our questionnaire treat no (n = 20/60, 33%) or up to five (n = 37/61, 61%) WD patients in an inpatient setting annually. Four departments reported between 6 and 10 patients annually. Material 3. Median and interquartile range (IQR; if applicable) for signs and symptoms in WD patients at the time of diagnosis in German university centers, subdivided into medical disciplines, as presented in Fig. 2. The IQR is shown as a range from Q1 (25%) to Q3 (75%). Material 4. Age distribution of WD patients at the time of diagnosis. Fifty-two departments provided data on age distribution (18 departments of pediatrics, 14 departments of neurology and 20 departments of gastroenterology). The majority (60%) of patients were older than 18 years at the time of diagnosis. In departments of neurology, 62% of patients were older than 35 years. Material 5. Application of the Leipzig score for WD diagnosis. Fifty-seven departments provided data on the Leipzig score (20 departments of pediatrics, 16 departments of neurology and 21 departments of gastroenterology). Fifty-one percent (n = 29/57) of all departments apply the Leipzig score. The proportion is higher in departments of gastroenterology (67%) than in departments of pediatrics (45%) or neurology (38%). Material 6. Median and interquartile range (IQR; if applicable) for diagnostic investigations for WD diagnosis at German university centers subdivided into medical disciplines, as presented in Fig. 3. The IQR is shown as a range from Q1 (25%) to Q3 (75%). [file 13023_2023_2731_MOESM1_ESM.docx]

## Additional file 1


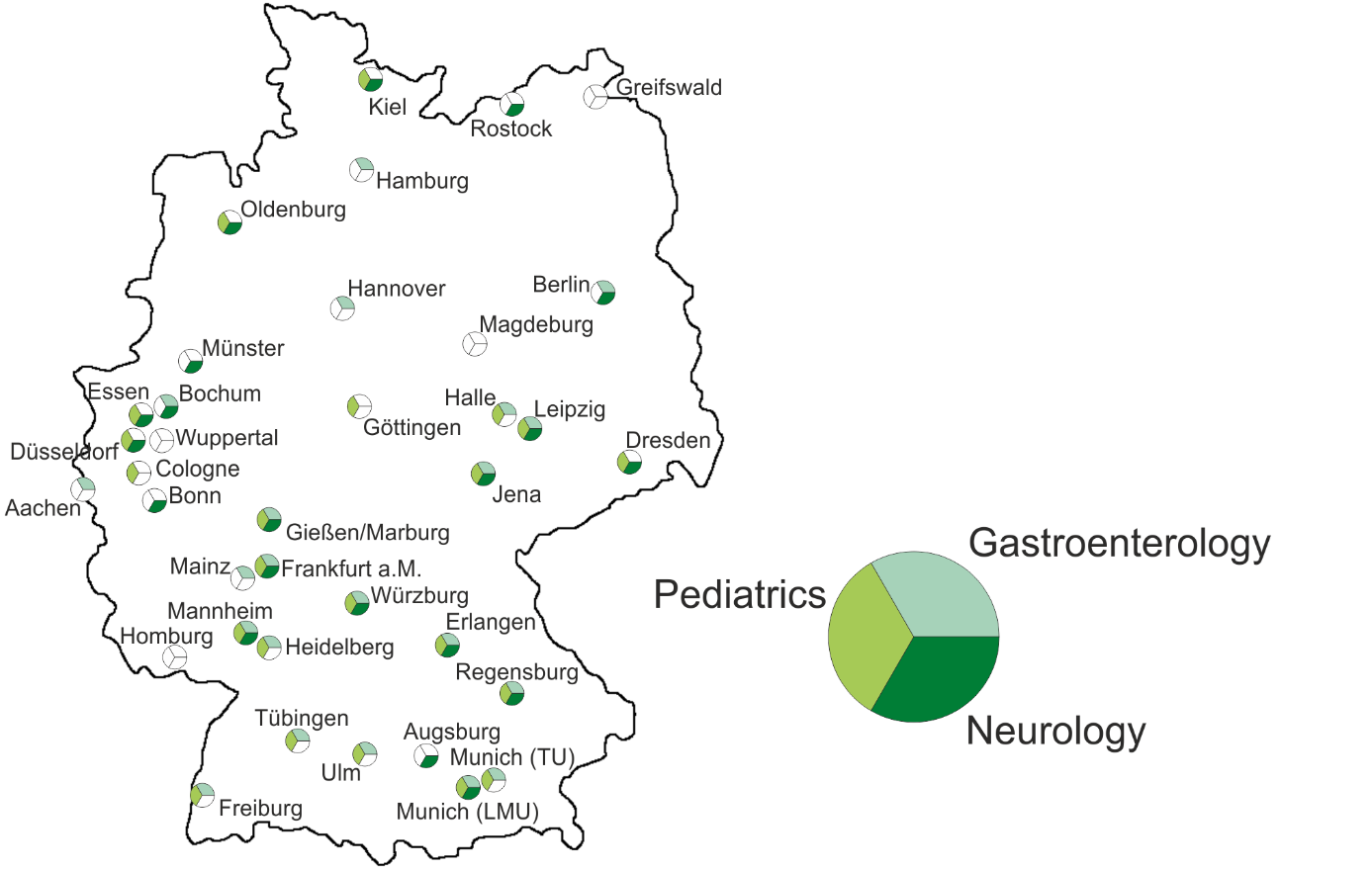


**Supplementary Material 1**: German university centers. Schematic map of Germany containing all centers that received our questionnaire (n = 108). The responding departments (n = 63/108, 58%) are highlighted by color for each discipline.


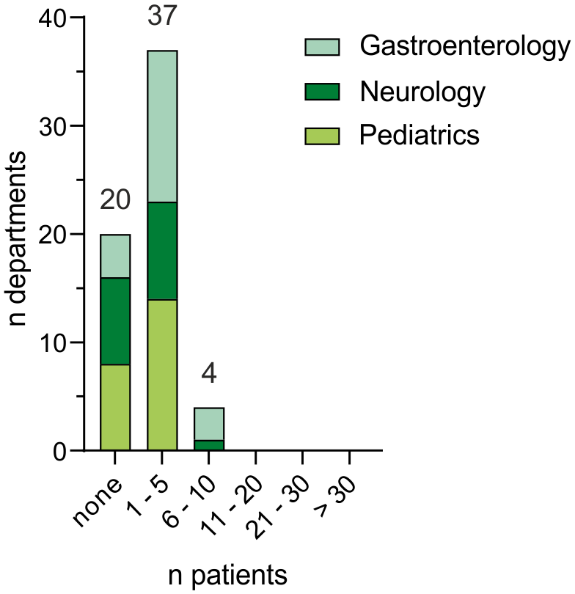


**Supplementary Material 2:** Number of patients with Wilson disease (WD) treated annually in an inpatient setting at German university centers subdivided into medical fields. Sixty-one departments provided data on the inpatient setting. Most departments returning our questionnaire treat no (n = 20/60, 33%) or up to five (n = 37/61, 61%) WD patients in an inpatient setting annually. Four departments reported between 6 and 10 patients annually.

|  | Pediatrics (n = 20) | | Neurology (n = 15) | | Gastroenterology (n = 21) | |
| --- | --- | --- | --- | --- | --- | --- |
|  | Median | IQR (Q1 - Q3) | Median | IQR (Q1 - Q3) | Median | IQR (Q1 - Q3) |
| hepatic symptoms | 100 | 80-100 | 50 | 10-90 | 90 | 61-100 |
| acute liver failure (ALF) | 0 | 0-9 | 0 |  | 5 | 0-17 |
| neurologic symptoms | 0 | 0-8 | 100 | 66-100 | 20 | 8-25 |
| psychiatric symptoms | 0 | 0-4 | 40 | 0-60 | 10 | 2-20 |
| hemolytic anemia | 0 | 0-4 | 0 |  | 5 | 0-15 |
| renal dysfunction | 0 |  | 0 |  | 1 | 0-13 |
| (cardio)myopathy | 0 |  | 0 |  | 0 | 0-5 |
| pancreatitis | 0 |  | 0 |  | 0 |  |
| asymptomatic | 0 | 0-20 | 0 | 0-10 | 10 | 0-19 |

**Supplementary Material 3:** Median and interquartile range (IQR; if applicable) for signs and symptoms in WD patients at the time of diagnosis in German university centers, subdivided into medical disciplines, as presented in Figure 2. The IQR is shown as a range from Q1 (25%) to Q3 (75%).


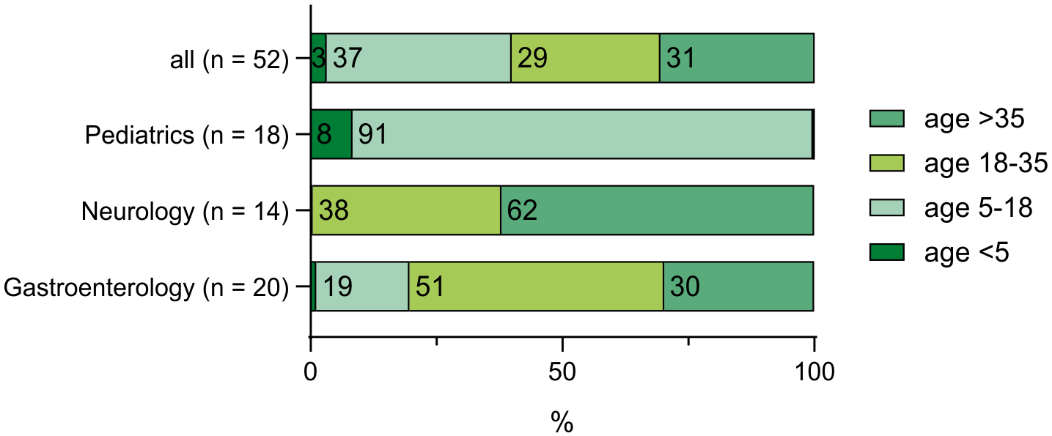


**Supplementary Material 4:** Age distribution of WD patients at the time of diagnosis. Fifty-two departments provided data on age distribution (18 departments of pediatrics, 14 departments of neurology and 20 departments of gastroenterology). The majority (60%) of patients were older than 18 years at the time of diagnosis. In departments of neurology, 62% of patients were older than 35 years.


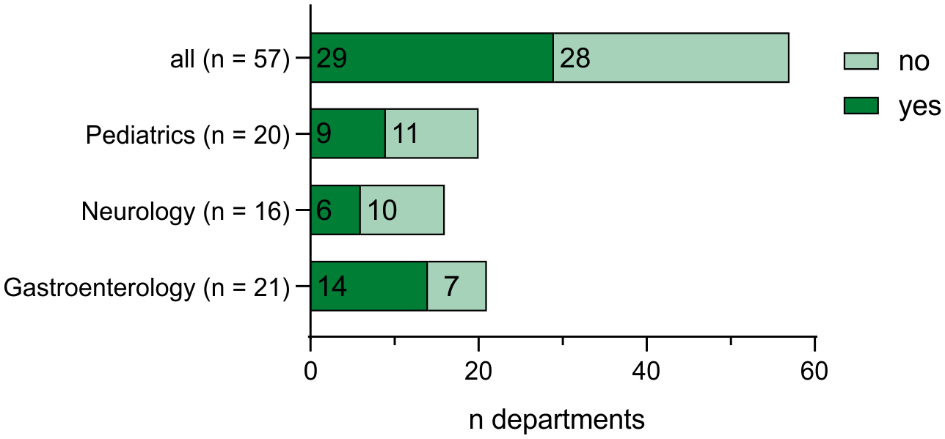


**Supplementary Material 5:** Application of the Leipzig score for WD diagnosis. Fifty-seven departments provided data on the Leipzig score (20 departments of pediatrics, 16 departments of neurology and 21 departments of gastroenterology). Fifty-one percent (n = 29/57) of all departments apply the Leipzig score. The proportion is higher in departments of gastroenterology (67%) than in departments of pediatrics (45%) or neurology (38%).

|  | Pediatrics (n = 20) | | Neurology (n = 14) | | Gastroenterology (n = 21) | |
| --- | --- | --- | --- | --- | --- | --- |
|  | Median | IQR (Q1 - Q3) | Median | IQR (Q1 - Q3) | Median | IQR (Q1 - Q3) |
| neurologic examination | 8 | 0-100 | 100 |  | 100 | 74-100 |
| cMRI | 0 | 0-41 | 100 |  | 43 | 16-100 |
| EEG | 0 |  | 6 | 0-100 | 12 | 0-26 |
| ophthalmologic split lamp examination | 100 |  | 100 | 75-100 | 100 | 98-100 |
| ceruloplasmin in sera | 100 |  | 100 |  | 100 |  |
| total copper in sera | 100 |  | 100 |  | 100 |  |
| calculated free copper in sera | 100 | 18-100 | 100 |  | 100 | 10-100 |
| measuring of free copper (CuEXC) | 0 | 0-45 | 0 |  | 0 | 0-20 |
| anemia diagnostics | 100 | 0-100 | 0 | 0-100 | 100 | 13-100 |
| copper excretion in 24 h urine | 100 |  | 100 |  | 100 |  |
| penicillamine challenging test | 75 | 6-100 | 0 | 0-23 | 10 | 0-40 |
| hepatic copper content in liver biopsy | 100 | 80-100 | 15 | 0-37 | 80 | 40-100 |
| histology in liver biopsy | 95 | 80-100 | 3 | 0-37 | 80 | 50-100 |
| *ATP7B* mutation analysis | 100 |  | 100 | 40-100 | 60 | 28-100 |

**Supplementary Material 6:** Median and interquartile range (IQR; if applicable) for diagnostic investigations for WD diagnosis at German university centers subdivided into medical disciplines, as presented in Figure 3. The IQR is shown as a range from Q1 (25%) to Q3 (75%).


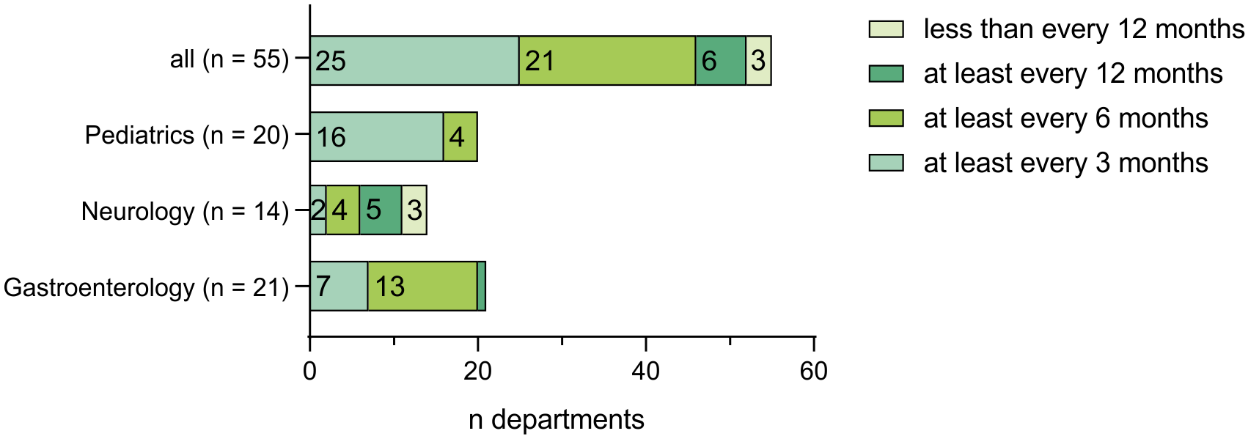


**Supplementary Material 7:** Frequency of routine monitoring for WD patients. Fifty-five departments provided data on the frequency of monitoring (20 departments of pediatrics, 14 departments of neurology and 21 departments of gastroenterology). Eighty-four percent (n = 46/55) of all departments see their patients at least biannually. Monitoring intervals tend to be shorter in departments of pediatrics (80% every three months, n = 16/20) than in departments of neurology (57% every 12 months or less, n = 8/14).


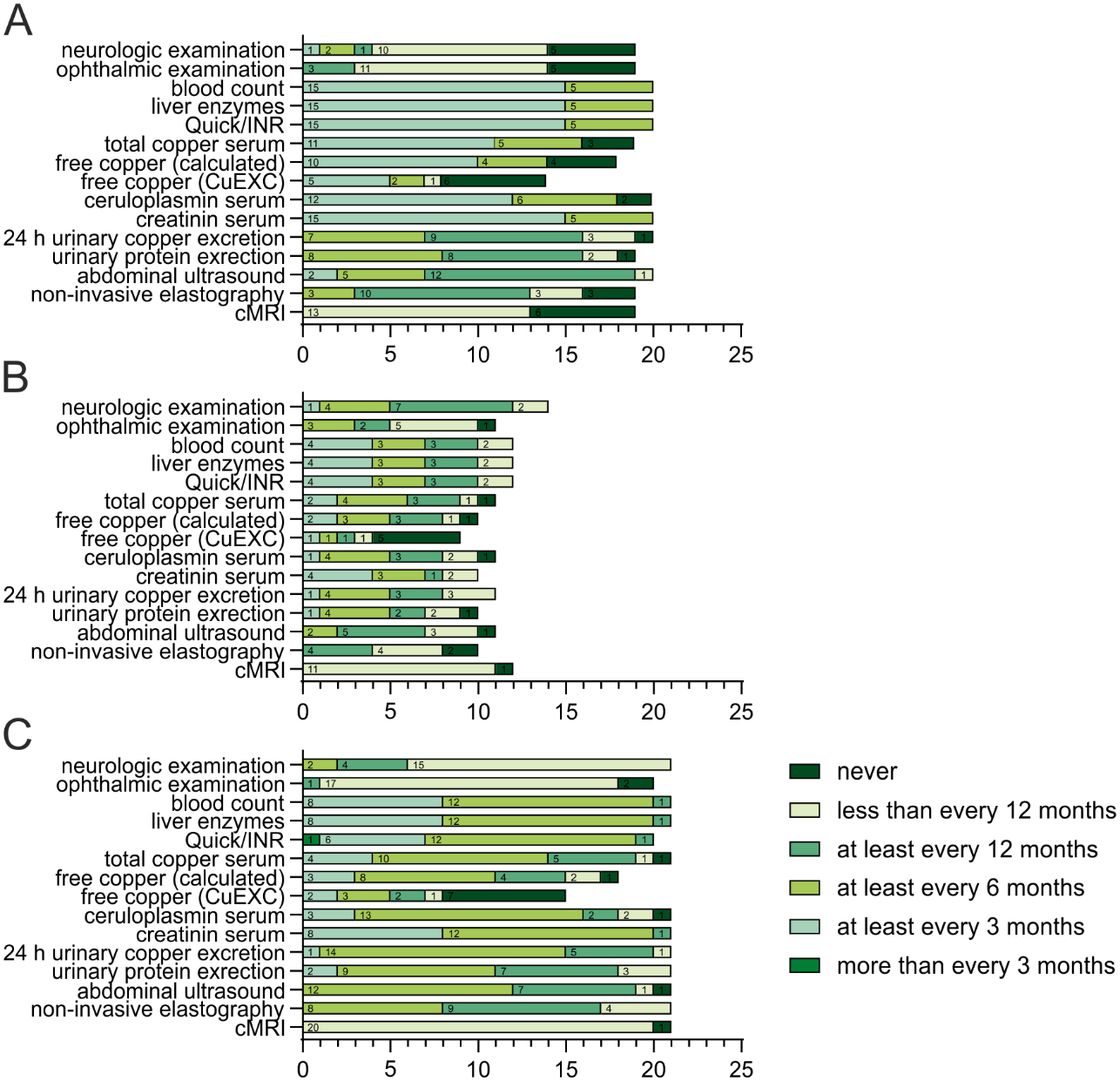


**Supplementary Material 8:** Frequency of monitoring investigations for WD patients in German university centers subdivided into disciplines. Up to 20 departments of pediatrics **(A)**, 14 departments of neurology **(B)** and 21 departments of gastroenterology **(C)** provided data on their application of certain monitoring investigations, depending on the procedure. Most investigations are performed at least annually or more often.


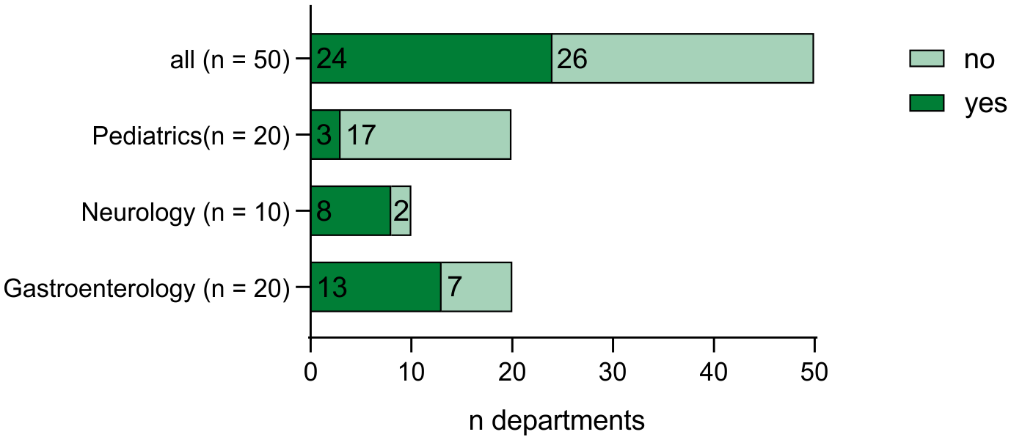


**Supplementary Material 9:** Cessation of chelator therapy before determination of 24-hour urinary copper excretion for monitoring in WD patients. Fifty departments provided data on the cessation of chelator therapy (20 departments of pediatrics, 10 departments of neurology and 20 departments of gastroenterology). Forty-eight percent (n = 24/50) pause the chelator therapy before the determination. The proportion was higher in departments of neurology and gastroenterology (together 70%, n = 21/30) than in pediatrics (15%, n = 3/20).


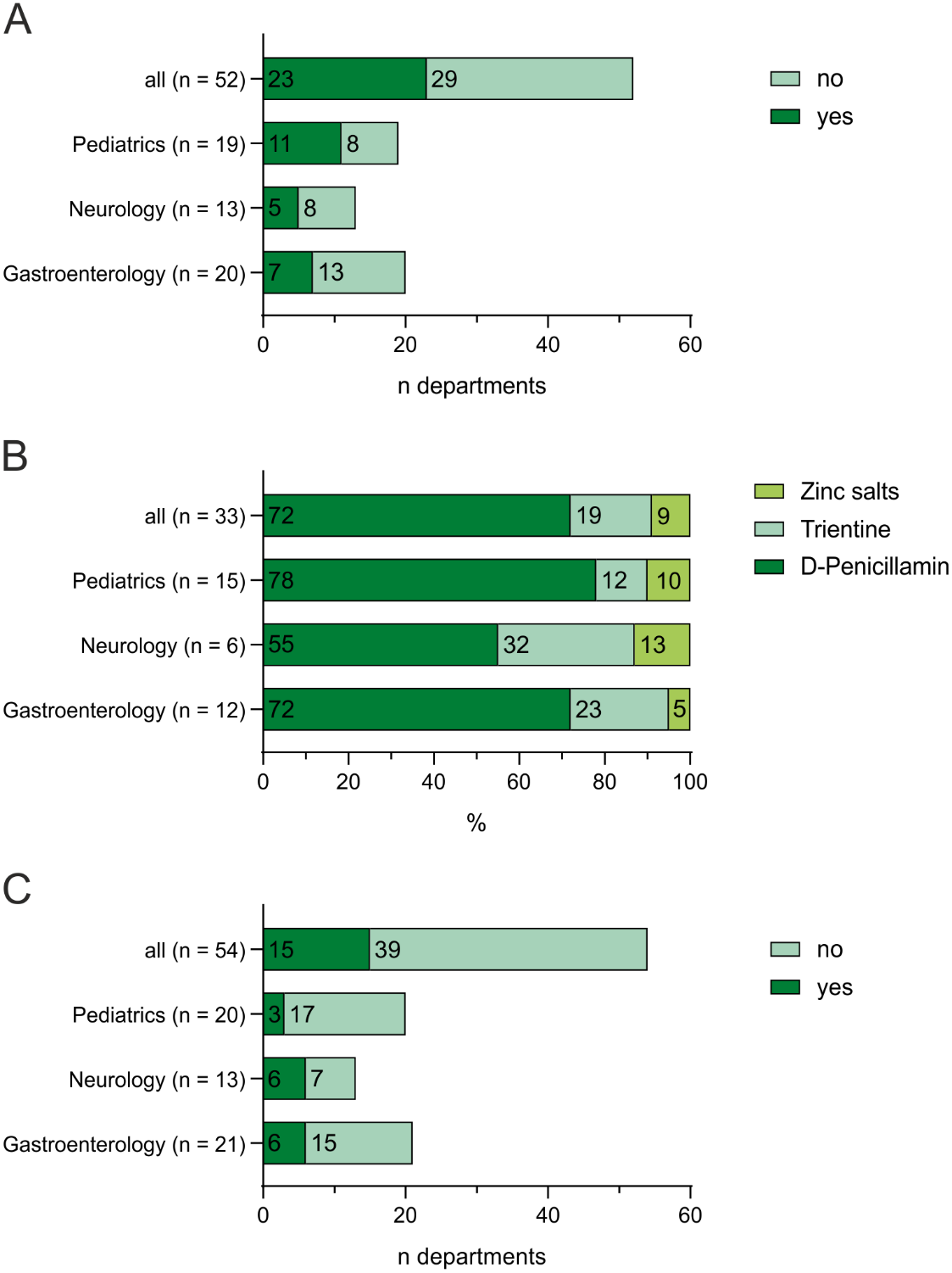


**Supplementary Material 10:** Medical therapy for WD patients. **(A)** Fifty-two departments provided data on zinc salt monotherapy (19 departments of pediatrics, 13 departments of neurology and 20 departments of gastroenterology). Zinc salt monotherapy is applied by 44% (n = 23/52) of all departments. The proportion was higher in departments of pediatrics (58%, n = 11/19) than in departments of neurology (38%, n = 5/13) and gastroenterology (35%, n = 7/20). **(B)** Thirty-three departments reported the usage of monotherapies alone (15 departments of pediatrics, 6 departments of neurology and 12 departments of gastroenterology). Regarding the use of chelator or zinc salt monotherapy in the departments, the mean in the departments was highest for D-penicillamine (72%). Trientine was less common (19%), and zinc salt monotherapy was rarely used (9%). **(C)** Fifty-four departments provided data on combination therapies (20 departments of pediatrics, 13 departments of neurology and 21 departments of gastroenterology). Zinc salt combination therapy was reported by 28% of the departments (n = 15/54).


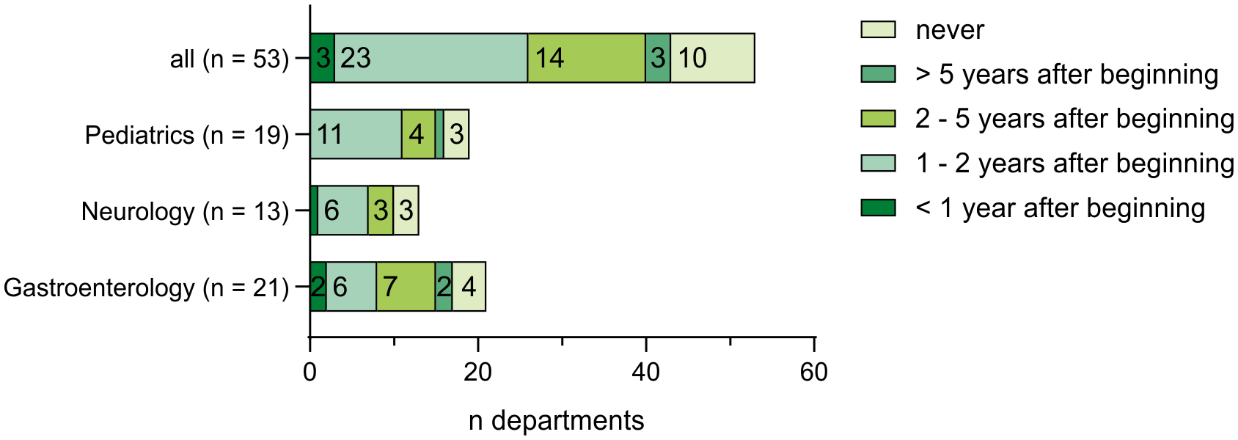


**Supplementary Material 11:** Time point of chelator therapy reevaluation in WD patients. Fifty-three departments provided data on therapy reevaluation (19 departments of pediatrics, 13 departments of neurology and 21 departments of gastroenterology). Eighty-one percent (n = 43/53) reevaluated the therapy, mostly after up to two years (49%, n = 26/53) or between two and five years (26%, n = 14/53). The evaluation of a chelator dose reduction after up to 2 years was more frequent in departments of pediatrics than in those of neurology and gastroenterology (58%, n = 11/19 vs. 44%, n = 15/34 in departments of neurology and gastroenterology taken together).


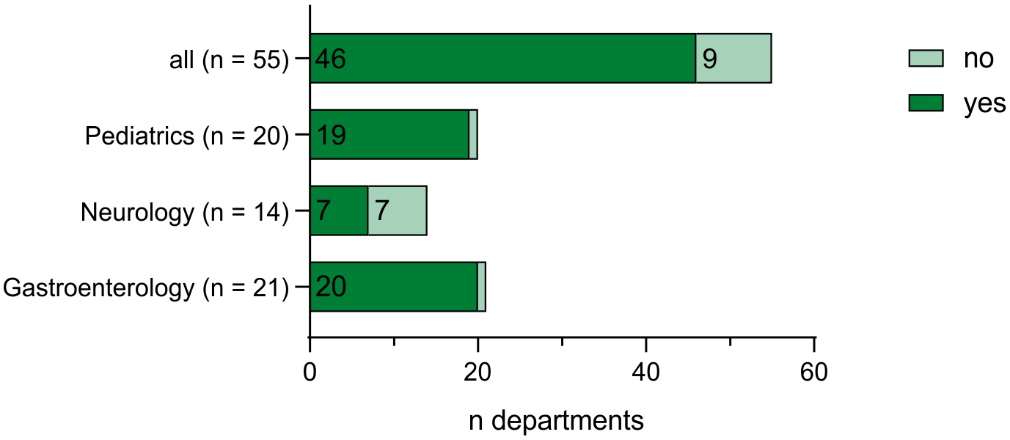


**Supplementary Material 12:** Family screening for relatives of WD patients. Fifty-five departments provided data on family screening (20 departments of pediatrics, 14 departments of neurology and 21 departments of gastroenterology). Eighty-four (n = 46/55) of all departments perform family screening on a regular basis. The proportion was lower in departments of neurology (50%, n = 7/14) than in departments of pediatrics and gastroenterology (taken together 95%, n = 39/41).


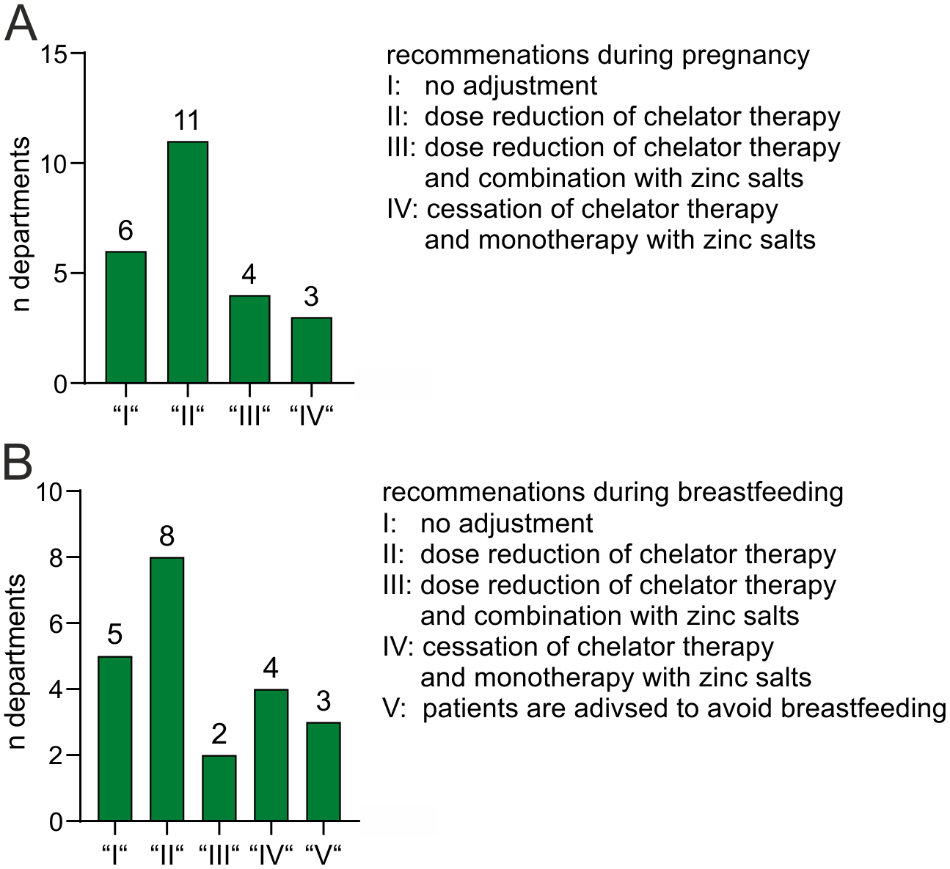


**Supplementary Material 13:** Recommendations for WD patients during pregnancy **(A)** and breastfeeding **(B)**. Twenty-four departments provided data on recommendations during pregnancy, and twenty-two departments provided recommendations during breastfeeding. Forty-six percent (n = 11/24) recommended a dose reduction during pregnancy, and 36% (n = 8/22) recommended a dose reduction during breastfeeding. In 25% (n = 6/24) and 23% (n = 5/22) of departments, respectively, there are no adjustments. Fourteen percent (n = 3/22) advised their patients to avoid breastfeeding.


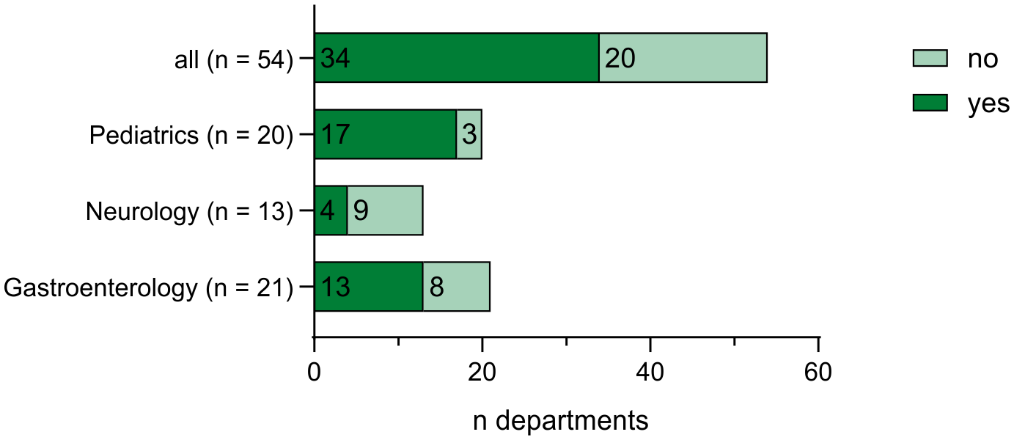


**Supplementary Material 14:** Nutrition counseling for WD patients. Fifty-four departments provided data on nutrition counseling (20 departments of pediatrics, 13 departments of neurology and 21 departments of gastroenterology). A total of 63% (n = 34/54) of departments offer nutrition counseling for WD patients in their departments. The proportion was higher in departments of pediatrics (85%, n = 17/20) and gastroenterology (62%, n = 13/21) than in departments of neurology (31%, n = 4/13).


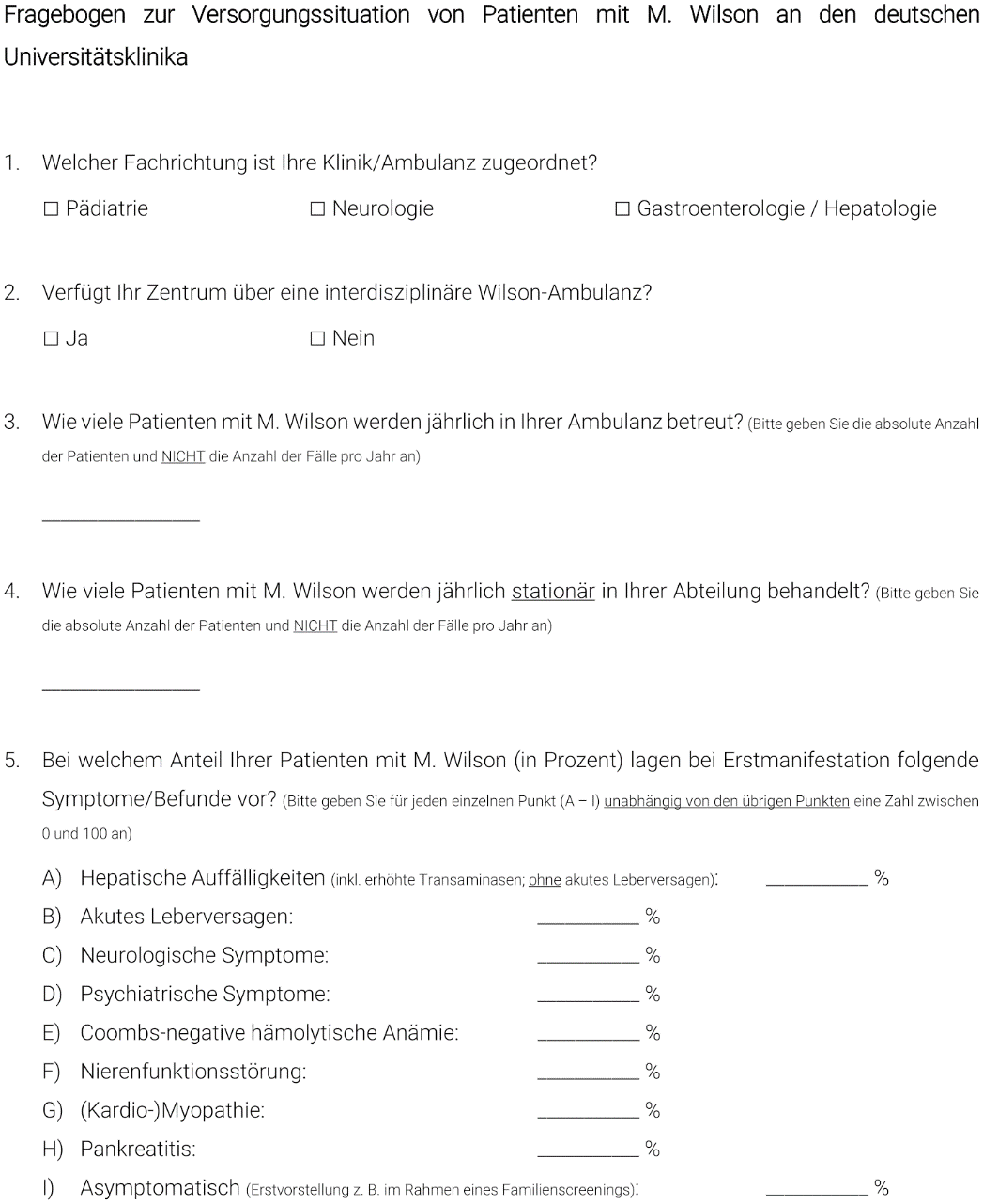

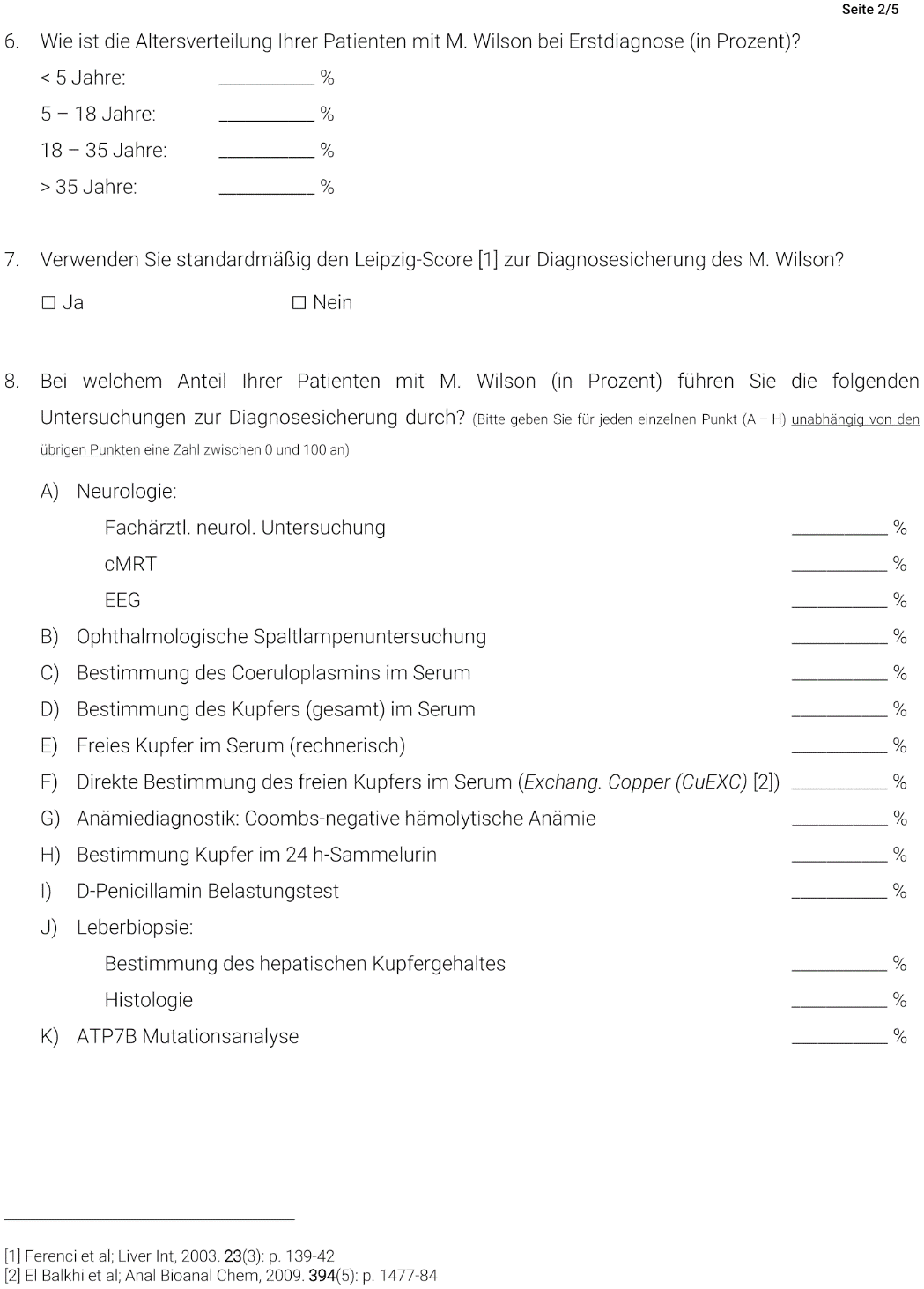

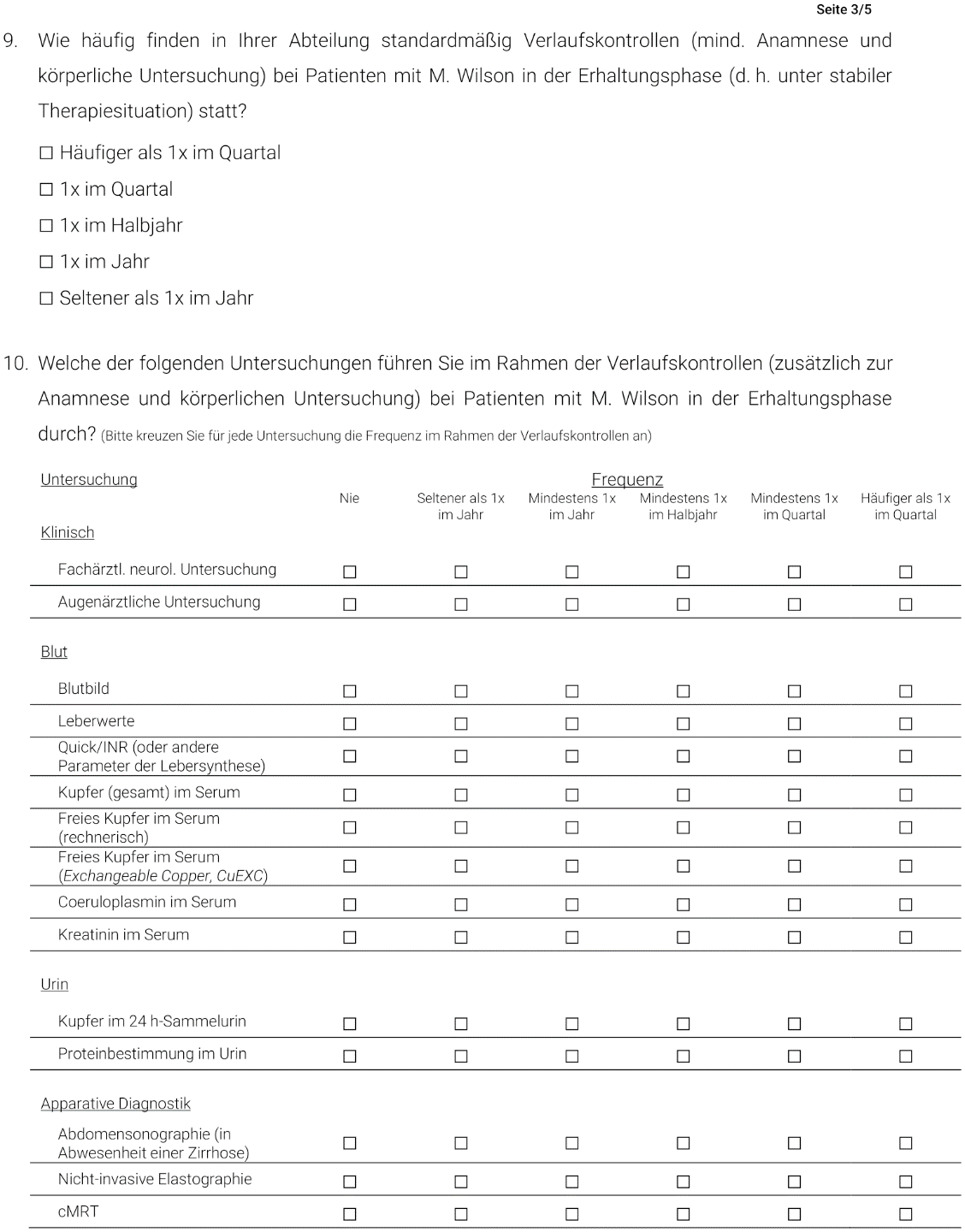

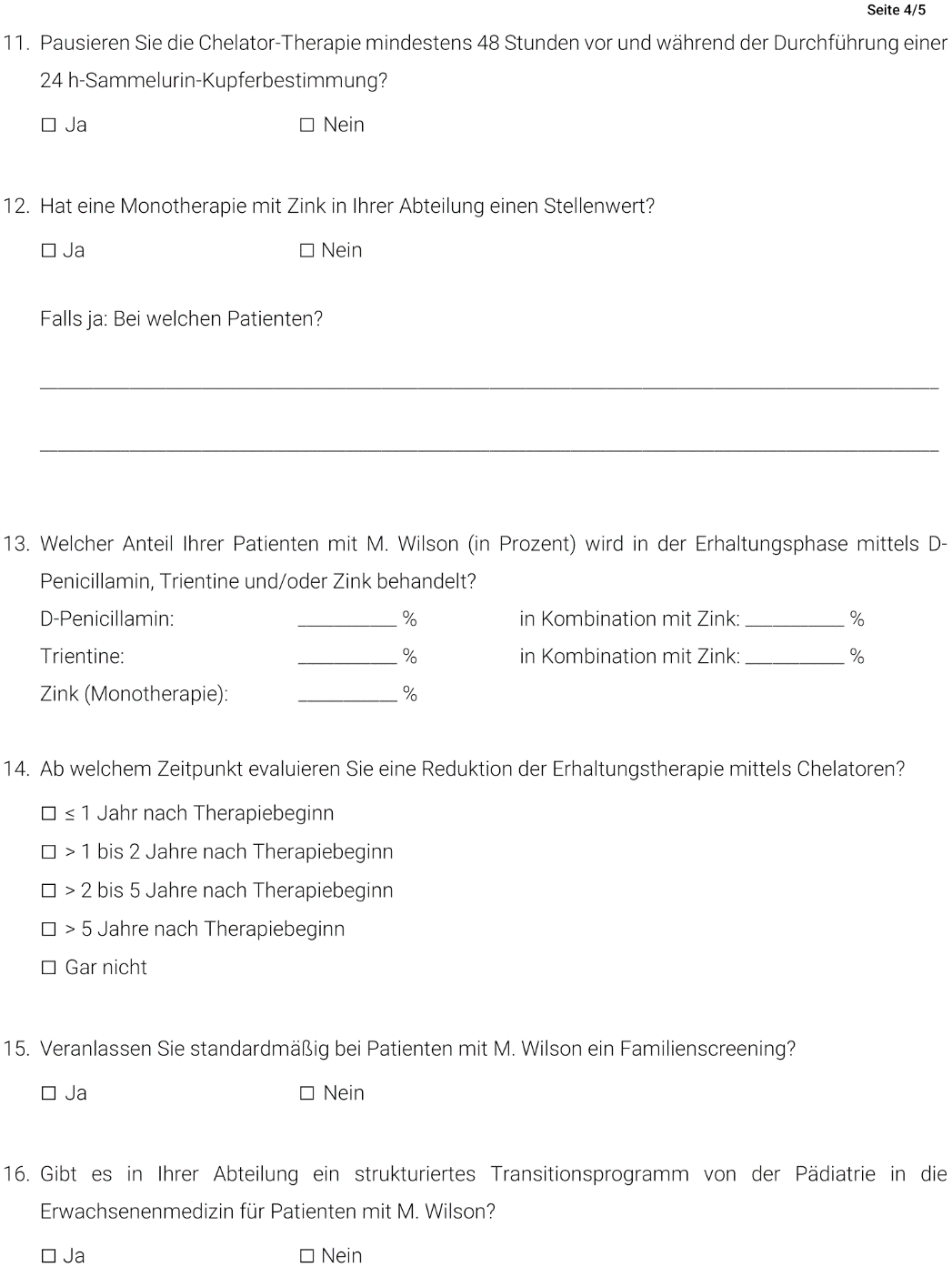

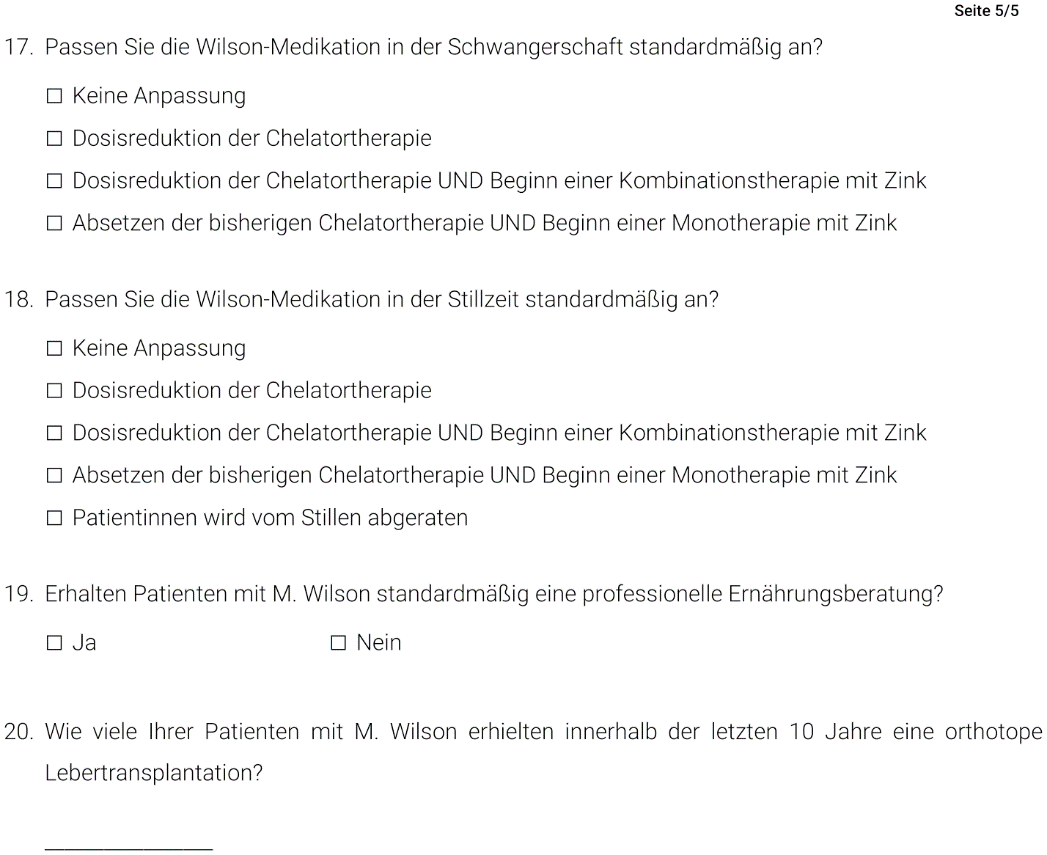


**Supplementary Material 15:** Original questionnaire.

| **No.** | **Question** |
| --- | --- |
|  | What is the medical discipline of your department? |
|  | Does your center have a multidisciplinary outpatient clinic? |
|  | How many patients are seen annually in your outpatient clinic? |
|  | How many patients are treated annually in an inpatient setting? |
|  | What proportion of your WD patients (in percent) had the following signs/symptoms at the time of disease manifestation? (hepatic symptoms, acute liver failure, neurologic symptoms, psychiatric symptoms, hemolytic anemia, renal dysfunction, (cardio-)myopathy, pancreatitis, asymptomatic) |
|  | What is the age distribution of WD patients in your department? |
|  | Do you use the Leipzig score for diagnosis on a regular basis? |
|  | In what proportion of WD patients (in percent) do you perform the following investigations for diagnosis? (neurologic examination, cMRI, EEG, ophthalmologic slit lamp examination, ceruloplasmin in sera, total copper in sera, calculated free copper in sera, measuring of free copper (CuEXC), anemia diagnostics, copper excretion in 24 h urine, penicillamine challenging test, hepatic copper content in liver biopsy, histology in liver biopsy, *ATP7B* mutation analysis) |
|  | How often do you perform follow-up examinations in WD patients in the maintenance phase (at least anamnesis and physical examination)? |
|  | What procedures do you perform during the follow-up of WD patients? (neurologic examination, ophthalmological examination, blood count, liver enzymes, Quick/INR, total copper serum, free copper (calculated), free copper (CuEXC), ceruloplasmin serum, creatinine serum, 24 h urinary copper excretion, urinary protein excretion, abdominal ultrasound, noninvasive elastography, cMRI) |
|  | Do you pause a chelator therapy 48 hours before the evaluation of 24 h urinary copper excretion? |
|  | Does zinc salt monotherapy have any relevance in your department? |
|  | What proportion of WD patients (in percent) is treated with D-penicillamine, trientine and/or zinc salts in the maintenance phase? |
|  | When do you reevaluate a reduction of chelator therapy during the maintenance phase? |
|  | Do you perform a family screening for WD patients on a regular basis? |
|  | Does your department have a structured transitional program from pediatrics to adult medicine? |
|  | Do you adjust the medication during pregnancy? (no adjustment, dose reduction of chelator therapy, dose reduction of chelator therapy and combination with zinc salts, cessation of chelator therapy and monotherapy with zinc salts) |
|  | Do you adjust the medication during breastfeeding? (no adjustment, dose reduction of chelator therapy, dose reduction of chelator therapy and combination with zinc salts, cessation of chelator therapy and monotherapy with zinc salts, patients are advised to avoid breastfeeding) |
|  | Do you provide professional nutrition counseling for WD patients? |
|  | How many WD patients in your department have undergone liver transplantation within the last decade? |

**Supplementary Material 16: Translation of the original questions.**

| **University Hospital**  **[alphabetically by city]** | **medical discipline** |
| --- | --- |
| Aachen | Gastroenterology |
| Augsburg | Neurology |
| Berlin | Gastroenterology |
|  | Neurology |
| Bochum | Neurology |
|  | Gastroenterology |
| Bonn | Neurology |
| Cologne | Pediatrics |
| Dresden | Gastroenterology |
|  | Pediatrics |
|  | Neurology |
| Düsseldorf | Neurology |
|  | Pediatrics |
| Erlangen | Neurology |
|  | Pediatrics |
|  | Gastroenterology |
| Essen | Pediatrics |
|  | Neurology |
| Frankfurt a. M. | Gastroenterology |
|  | Neurology |
|  | Pediatrics |
| Freiburg | Pediatrics |
|  | Gastroenterology |
| Gießen/Marburg | Neurology |
|  | Pediatrics |
|  | Gastroenterology |
| Göttingen | Pediatrics |
| Halle | Gastroenterology |
|  | Pediatrics |
| Hamburg | Gastroenterology |
| Heidelberg | Gastroenterology |
|  | Pediatrics |
| Jena | Neurology |
|  | Pediatrics |
|  | Gastroenterology |
| Kiel | Pediatrics |
|  | Neurology |
| Leipzig | Pediatrics |
|  | Gastroenterology |
|  | Neurology |
| Mainz | Gastroenterology |
| Mannheim | Neurology |
|  | Pediatrics |
|  | Gastroenterology |
| Munich (LMU) | Pediatrics |
|  | Neurology |
|  | Gastroenterology |
| Munich (TU) | Gastroenterology |
|  | Pediatrics |
| Münster | Neurology |
| Oldenburg | Neurology |
|  | Pediatrics |
| Regensburg | Neurology |
|  | Gastroenterology |
|  | Pediatrics |
| Rostock | Neurology |
| Tübingen | Pediatrics |
|  | Gastroenterology |
| Ulm | Pediatrics |
|  | Gastroenterology |
| Würzburg | Neurology |
|  | Pediatrics |
|  | Gastroenterology |

**Supplementary Material 17:** Institutions participating in our survey, listed alphabetically, by medical discipline.
